# A multi-fingerprint browser for the ZINC database

**DOI:** 10.1093/nar/gku379

**Published:** 2014-04-29

**Authors:** Mahendra Awale, Jean-Louis Reymond

**Affiliations:** Department of Chemistry and Biochemistry, University of Berne, Freiestrasse 3, Berne-3012, Switzerland

## Abstract

To confirm the activity of an initial small molecule ‘hit compound’ from an activity screening, one needs to probe the structure–activity relationships by testing close analogs. The multi-fingerprint browser presented here (http://dcb-reymond23.unibe.ch:8080/MCSS/) enables one to rapidly identify such close analogs among commercially available compounds in the ZINC database (>13 million molecules). The browser retrieves nearest neighbors of any query molecule in multi-dimensional chemical spaces defined by four different fingerprints, each of which represents relevant structural and pharmacophoric features in a different way: sFP (substructure fingerprint), ECFP4 (extended connectivity fingerprint), MQNs (molecular quantum numbers) and SMIfp (SMILES fingerprint). Distances are calculated using the city-block distance, a similarity measure that performs as well as Tanimoto similarity but is much faster to compute. The list of up to 1000 nearest neighbors of any query molecule is retrieved by the browser and can be then clustered using the K-means clustering algorithm to produce a focused list of analogs with likely similar bioactivity to be considered for experimental evaluation.

## INTRODUCTION

Small molecule drug discovery relies on the identification and iterative optimization of bioactive compounds considering one or several activity and property parameters (1,2). Once an initial active compound, a so-called hit, has been identified, its optimization requires to evaluate close structural analogs (3,4). One particularly straightforward first step in this optimization should consist in acquiring any commercially available compounds having a relevant structural similarity to the hit, whereby the structural similarity can be quantified using various ligand-based virtual screening (LBVS) methods (5–7), in particular those based on comparing molecular fingerprints (8) using similarity measures (9,10). This approach is particularly relevant today because over 13 million different drug-like molecules are commercially available and collected in a common database ZINC (11). However, the current options to search this database, such as the similarity search function at the ZINC website, or other database browsers (12–15) and visualization tools, such as the MQN (molecular quantum number) and SMIfp (SMILES fingerprint) browsers and mapplets (16,17), only offer limited capabilities in terms of selecting different fingerprint types and assembling a focused library of analogs of a given hit compound.

The steps necessary to perform a relevant selection of analogs of a particular hit compound in ZINC are more complex than a simple similarity search. First, one needs to probe the existence of hit analogs by examining similarities along different aspects of molecular structure such as pharmacophores (18–20), molecular shape (21–24), substructures (25,26) or other molecular descriptors known to be good predictors of biological activity (27–32). Second, one must also analyze the resulting list of closest analogs by grouping similar molecules together using clustering (12,33,34), such as to assemble a cost-effective, focused yet diverse list of analogs. This search and clustering routine should be fast and easy to use by experts and non-experts with minimal requirement for computational infrastructure.

### Multi-fingerprint browser

The multi-fingerprint browser presented here (http://dcb-reymond23.unibe.ch:8080/MCSS/) provides an intuitive user interface with a simple workflow to rapidly identify close analogs of a query molecule among commercially available compounds in the ZINC database (11). The browser provides an array of options for formulating the query and allows for the visualization/analysis of the virtual screening (VS) results with k-mean clustering. The search engine of the multi-fingerprint browser uses the city-block distance (CBD) to rank the compounds in decreasing order of similarity to the input query molecule. The similarity search space can be constructed from four different fingerprints, each of which represents relevant structural and pharmacophoric features in a different way. Two of them are binary fingerprints, namely a daylight-type substructure fingerprint (sFP) and an extended connectivity fingerprint (ECFP4) (35), which describe substructures of the molecule in the form of bit wise vectors where ‘1’ or ‘0’ indicates the presence or absence of a particular substructure (25). sFP and ECFP4 are well-established fingerprints which are used widely for VS. The other two property spaces are derived from scalar fingerprints, namely MQNs (36), featuring 42 integer value descriptors counting atoms, bonds, polar groups and topological features (Supplementary Table S1), and the SMIfp (17), featuring the counts of 34 different characters in the SMILES representation of a molecule (Supplementary Table S2). Both MQN and SMIfp were recently developed in our group and have been shown to provide reference feature spaces with capability for LBVS and visualization of large databases (16,37). More importantly, MQN and SMIfp provide a way to identify new chemotypes by similarity searching because they do not search for the exact substructure information. The multi-fingerprint browser presented here extends our previously reported MQN and SMIfp browsers by newly including the sFP and ECFP4 CBD similarity search, and adds the clustering option as a new functionality.

## MATERIALS AND METHODS

### Processing and organization of ZINC

ZINC is an open-access database of commercially available small organic molecules for drug discovery and currently contains more than 13 million unique compounds (11). A new version of the ZINC database is released periodically with updated information for molecules and vendors. Accordingly, we have planned to update the compound library in the multi-fingerprint browser every 6 months. For construction of the browser, molecules from ZINC were processed in SMILES format using an in-house-built java program utilizing the Java Chemistry library (JChem) from ChemAxon, Pvt Ltd. Counter ions were removed and the ionization state of the molecules was adjusted to pH 7.4. Each of the ZINC molecules was annotated with its molecular formula, count of hydrogen-bond acceptors (HBAs), hydrogen-bond donors (HBDs) and the numbers of oxygen and nitrogen atoms. MQN and SMIfp were calculated using our previously reported source codes written in Java. For sFP and ECFP4, 1024-bit hash fingerprints were calculated using the JChem library. During fingerprint calculation, the path length (in sFP) was set to 7 and the bond diameter (in ECPF4) was set to 4. Subsequently, the ZINC database was organized in the form of hash tables (one for each fingerprint space), where the hash key is defined as the sum of all bit values in the fingerprint (total sum). This pre-organization is the key to enable fast searching by CBD, because it allows one to confine the search to subsections of the database matching the hash key of the query molecule within a specified distance (CBD) limit. For example, when the query molecule's fingerprint has the total sum of 100 and the goal is to find nearest neighbors within distance of CBD ≤ 10, one has to only look in part of the hash table with hash key values in the range of 90 ≤ total sum ≤ 110 [for details see (38)].

### Similarity metrics

The city-block distance between two points (CBD*_A, B_*), *A* and *B*, with *K* dimensions is calculated as

\begin{equation*} {\rm CBD}_{A,B} = \sum\limits_{j = 1}^K {|A_j - B_j |}. \end{equation*}

For molecules *A* and *B* represented by vectors *X_A_* and *X_B_* with length *n* and attributes *j*, their Tanimoto similarity coefficient (*T_A, B_*) is calculated as

\begin{equation*} T_{A,B} = \frac{{\sum\limits_{j = 1}^n {X_{jA} \cdot X_{jB} } }}{{\sum\limits_{j = 1}^n {\left( {X_{jA} } \right)^2 + \sum\limits_{j = 1}^n {\left( {X_{jB} } \right)^2 - \sum\limits_{j = 1}^n {X_{jA} \cdot X_{jB} } } } }}. \end{equation*}

### Benchmarking similarity search methods

**Figure 1.**
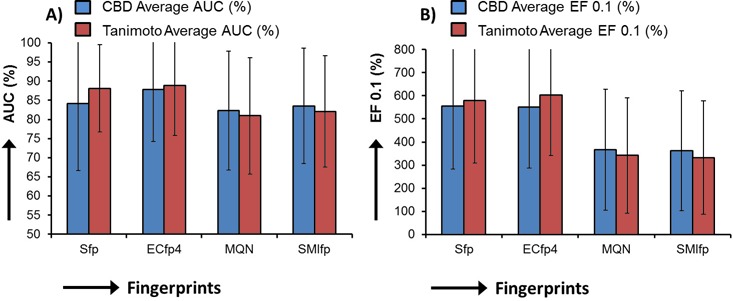
Average AUC values (**A**) and EF at 0.1% of screened database (**B**), for recovery of 40 sets of actives in the directory useful decoys (DUD) from the ZINC database by using CBD_fingerprint_ (blue bars) and *T*_fingerprint_ (brown bars) as scoring functions. Receiver operating characteristic curves (ROC) are provided in Supplementary Figures S1 and S2. ROC curves, average AUC and EF at 1% for recovery of DUD actives from the corresponding set of DUD decoys are provided in Supplementary Figures S3–S5.

The efficiency of a similarity search method is typically judged by its ability to recall known active compounds from a background noise database (decoys). The sFP, ECFP4, MQN and SMIfp fingerprints were evaluated for recovery of active compounds of 40 target proteins from their corresponding decoys available from the Directory of Useful Decoys (DUD; data provided in Supplementary Figures S3–S5) and from the entire ZINC database (39). The enrichment results against entire ZINC are represented as average of Area Under receiver operating characteristic Curves (AUC) (Figure 1, Supplementary Figures S1 and S2) and average of enrichment factors at 0.1% (EF 0.1) of screened database. MQN and SMIfp show comparable performance for the recovery of various bioactivity classes, although they do not match sFP/ECFP4 performance. The superior performance of sFP and ECFP4 can be partly explained by the fact that decoys were selected for low substructure similarity to the actives. Furthermore, switching the scoring function from city-block distance (CBD_fingerprint_) to Tanimoto coefficient (*T*_fingerprint_) shows no significant change in recall of actives from decoys. The Tanimoto coefficient is a widely recognized similarity metric for binary substructure fingerprints (sFP and ECFP4). For the multi-fingerprint browser, our choice was to use CBD_fingerprint_ because it can be computed as fast as the Tanimoto coefficient, but additionally it allows for an efficient pre-organization of the database for similarity searching.

## DEFINITION OF QUERY AND SETTING SEARCH PARAMETERS

**Figure 2.**
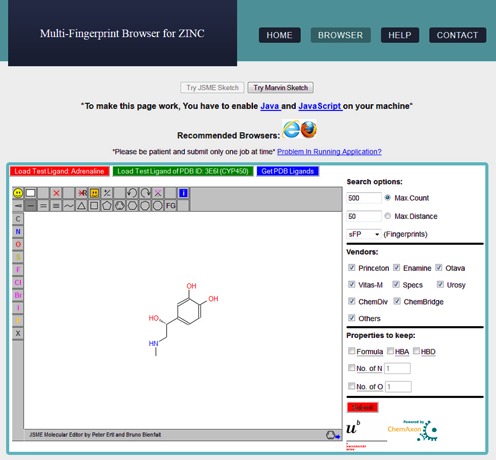
Query page of Multi-Fingerprint browser for setting up search parameters. Search options can be divided into four parts: (i) molecular drawing panel for input query molecule, structure is shown for adrenaline; (ii) selection of one of the four fingerprint spaces (sFP/ECFP4/MQN/SMIfp) and of Max Count or Max Distance mode; (iii) choice of specific vendors for the search (by default all vendors will be searched); (iv) filters to fix certain molecular properties of the query molecule.

The graphical user interface (GUI) of the multi-fingerprint browser loads with the initial web page, which provides several options for formulation of a query (Figure 2). Search options can be broadly grouped into four parts, each of which is discussed below.

- **Input molecule**
  The JSME molecular editor from Peter Ertl *et al.* (40) and MarvinSketch from ChemAxon Pvt Ltd are provided as two options to input the query molecule for similarity searching. The query structure can be drawn, or the molecule can be pasted as smiles/mol2/sdf format in the molecular editors. JSME\MarvinSketch editors are embedded in the HTML page as Java applets, which demands active JavaScript and Java plugin (version ≥1.6) in the client web browser. Additionally, an option is available to extract the query molecule from the Protein Data Bank (PDB) using the PDB ID of the protein–ligand complex of interest. The PDB ligand data were downloaded from http://ligand-expo.rcsb.org/ website and stored on web server, which will be updated periodically (every 6 months).
- **Search method**
  First, one of the fingerprint spaces (sFP, ECFP4, MQN or SMIfp) must be selected for similarity searching from the drop down menu. Next, the search specification Max Count (in number of molecules) or Max Distance (in CBD_fingerprint_) must be enabled by selection of the respective radio button, using either the default value or a user-specified value, whereby the Max Count cannot exceed 1000 compounds. Upon submission of the query, the search engine of the multi-fingerprint browser then searches the hash table files in order of increasing difference in total sum of the query molecule until one of the Max Count or Max Distance criteria has been reached.
- **Choice of vendors**
  Criteria can be set to retrieve nearest neighbors either from all the vendors (>150) available in the ZINC database or from any possible combination of nine vendors: Princeton, Enamine, Otava, Vitas-M, Specs, Urosy, ChemDiv, ChemBridge and Others (all other remaining vendors). The listed vendors are our own choice and are major contributors to the ZINC database. By default, the multi-fingerprint browser retrieves compounds from all vendors. The choice of vendors can be specified by selection of appropriate check boxes.
  Searching in the vendor space is enabled by using bit mask values to store the vendor information of the molecule. A bit mask is an integer number encoding the information for ON (1) and OFF (0) bits in underlying binary equivalent. Bits were assigned to each of the nine vendors. Depending upon availability of vendors, specific bits were turned ON and the corresponding bit mask value was generated and stored for each of the database molecules. During similarity searching, the choice of vendors made by the user is defined as ‘wanted bit mask’ and searched inside the database using Bitwise OR operation.
- **Molecular property filters**
  Nearest neighbors can be requested to have certain molecular properties in common. For instance, locking the molecular formula option extracts compounds that are at least formula isomers of the input query molecule. Knowing the importance of HBA and HBD atoms for the interaction of small molecules with their target proteins, options are provided to retain HBA and HBD atom counts of the input query molecule in nearest neighbors. Furthermore, the atomic composition of the compounds can be tweaked by specifying the desired number of oxygen and nitrogen atoms. The use of property filters usually increases the search time of the ‘Max Count’ mode because the search might have to go through many more hash tale entries to reach the preset number of molecules.
  Once all the parameters are set, a maximum of 1000 nearest neighbors of the input query molecule can be retrieved by clicking on the ‘Submit’ button. Typically the execution of a search takes a few seconds to a few minutes. Imposing more restrictive criteria on nearest neighbors leads to a considerable increase in search time. Similarity searches in MQN and SMIfp spaces are usually much faster than in sFP and ECFP4 spaces due to a smaller number of dimensions and a more efficient organization of the database.

## RESULTS

Search results are exemplified with searching for 500 nearest neighbors of 4, 5-β-trihydroxy-*N*-methylphenethylamine (Adrenaline/Epinephrine) in MQN fingerprint space.

### Visualization of nearest neighbors

**Figure 3.**
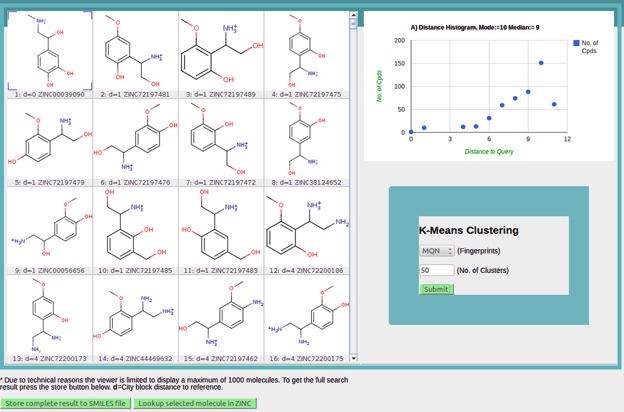
Similarity search results for retrieval of 500 nearest neighbors of adrenaline in MQN space. Structures of nearest neighbors are shown in the molecule table built with the MarvinView Applet from ChemAxon Pvt Ltd. The scatter plot showing the number of compounds as a function of CBD to the query is constructed with the ‘Google Chart’ application. These nearest neighbors can be saved to a file (green button at bottom of page) or can be further analyzed by clustering using K-means algorithm.

The structures of nearest neighbors retrieved by the search engine are displayed in a 4xn molecular table built with the MarvinView Applet provided by ChemAxon Pvt Ltd (Figure 3). Nearest neighbors are sorted by increasing CBD to the query molecule. A quick overview of the similarity search results is provided in the scatter plot at right showing the count of nearest neighbors as a function of CBD_fingerprint_ to the query molecule. As observed from the scatter plot, CBD for adrenaline analogs ranges from 0 to 11 with the maximum occurrence of compounds at distance 10. These nearest neighbors show overall similar composition in terms of ring, atom types and functional groups compared to adrenaline. Each of the displayed molecules is tagged with the ZINC id and can be linked to the parent ZINC database website to acquire detailed information on the molecule.

Comparative analysis of the CBD-nearest neighbors of adrenaline in four fingerprint spaces shows that analogs provided by MQN or SMIfp are considerably different from those retrieved by sFP and ECPF4 similarity search. The sFP and ECFP4 analogs mostly preserve the substructure pattern of adrenaline: phenyl ring with 1-hydroxy-2-(methylamino) ethyl substituent at position 4. This is particularly important when the basis is to study structure–activity relationship of the lead molecule. On the other hand, the rearrangement of the hydroxyl, amino and other groups proposed by the MQN and SMIfp searches suggests substructure patterns that are substantially different from the input query molecule, a feature desirable for the identification of new chemotypes.

### Clustering of nearest neighbors

It is important to examine the initial list of nearest neighbors for redundancy and structural diversity (41,42). This knowledge can then be used to construct a focused, cost-effective, more representative and diverse chemical library with increased likelihood to find bioactive compounds. To assist in this task, the multi-fingerprint browser provides a way to group the nearest neighbors using the well-known K-means clustering algorithm. Compounds can be grouped into a predefined number of clusters using one of the four similarity measures (Figure 3). Note that nearest neighbor searching and clustering are two separate steps and different fingerprints may be used in each step.

**Figure 4.**
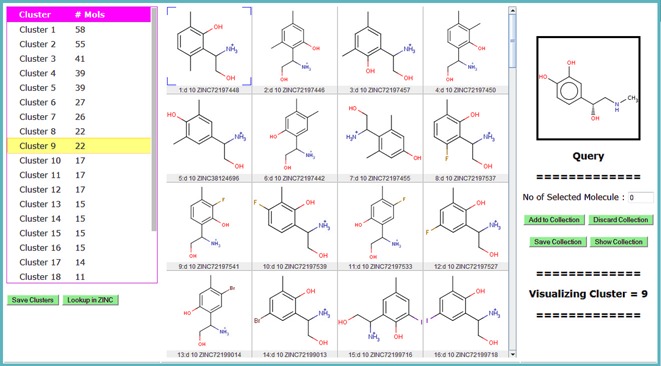
Visualization/analysis interface for clustering results. The list of 50 clusters for MQN analogs of adrenaline is shown in the table on the left. The molecular table on the right displays the structures of compounds in cluster no. 9, which is selected in the table on the left. The centroid of the cluster is displayed at position 1 in the table. The list of clusters can be saved to a file for further analysis using the ‘Save Clusters’ button. Molecules from the clusters can be selected manually and saved to file using ‘Add to Collection’ and ‘Save Collection’ buttons, respectively.

The clustering results obtained for adrenaline analogs in MQN space (‘number of clusters’ parameter was set to 50) are shown in Figure 4. The table on the left shows the list of clusters ordered according to decreasing size. Apart from a few small groups, clusters are rather uniformly populated in this example, although this is not always the case. Visualization of the various clusters of adrenaline analogs shows that they feature different families of compounds. For example, cluster no. 9 contains trisubstituted benzene rings with minor modifications of functional groups. The ‘Centroid’ of the cluster is displayed at the first position in the molecular table and can be used as ‘cluster representative’ for the final selection. Note that the centroid is not necessarily the best cluster representative and that clusters sometimes contain diverse compounds, in which case the selection of more than one compound may be necessary.

### Saving the results

The list of nearest neighbors obtained from the similarity search can be saved to a file. This file contains the SMILES representation of the molecules, their ZINC id and CBD to the input query molecule in the fingerprint space used for searching. The same list can be saved after clustering, in which case molecules are grouped by cluster and annotated additionally with their cluster number. This file can be used later for further analysis/visualization using any molecular viewer.

## CONCLUSIONS

With its intuitive GUI, the multi-fingerprint browser features a practical and versatile similarity search tool for the ZINC database. This browser can be readily used in hit identification or lead optimization and provides a valuable source of information for medicinal chemists and other researchers in the drug discovery field.

## SUPPLEMENTARY DATA

Supplementary Data are available at NAR Online.

Supplementary Data
