# Supplementary material for: A multi-fingerprint browser for the ZINC database
Source: Nucleic Acids Res. 2014 Apr 29;42(Web Server issue):W234–9. doi: 10.1093/nar/gku379 (PMC4086083; doi:10.1093/nar/gku379)
Supplement: Supplementary Data [file supp_42_W1_W234__index.html]

Supplementary Data 

# A multi-fingerprint browser for the ZINC database

## Supplementary Data

**Files in this Data Supplement:**

- SUPPLEMENTARY DATA
