# Supplementary material for: A multi-fingerprint browser for the ZINC database
Source: Nucleic Acids Res. 2014 Apr 29;42(Web Server issue):W234–9. doi: 10.1093/nar/gku379 (PMC4086083; doi:10.1093/nar/gku379)
Supplement: Supplementary Data [file supp_gku379_nar-00251-web-b-2014-File002.doc]

Supporting Information for:

**A Multi-Fingerprint Browser for the ZINC Database**

Mahendra Awale and Jean-Louis Reymond^*^

Department of Chemistry and Biochemistry, University of Berne, Freiestrasse 3, Berne-3012, Switzerland

*To whom correspondence should be addressed. Tel: +41 31 631 43 25; Fax: +41 31 631 80 57; Email: [jean-louis.reymond@dcb.unibe.ch](mailto:jean-louis.reymond@ioc.unibe.ch)

**Table S1**. The 42 molecular quantum numbers (MQNs)

| **Atom counts** (12) | | **Bond counts** (7) | |
| --- | --- | --- | --- |
| c | carbon | asb | acyclic single bonds |
| f | fluorine | adb | acyclic double bonds |
| cl | chlorine | atb | acyclic triple bonds |
| br | bromine | csb | cyclic single bonds |
| i | iodine | cdb | cyclic double bonds |
| s | sulphur | ctb | cyclic triple bonds |
| p | phosphorous | rbc | rotatable bond count |
| an | acyclic nitrogen |  |  |
| cn | cyclic nitrogen | **Topology counts^b)^** (17) | |
| ao | acyclic oxygen | asv | acyclic monovalent nodes |
| co | cyclic oxygen | adv | acyclic divalent nodes |
| hac | heavy atom count | atv | acyclic trivalent nodes |
|  |  | aqv | acyclic tetravalent nodes |
| **Polarity counts^a)^** (6) | | cdv | cyclic divalent nodes |
| hbam | H-bond acceptor sites | ctv | cyclic trivalent nodes |
| hba | H-bond acceptor atoms | cqv | cyclic tetravalent nodes |
| hbdm | H-bond donor sites | r3 | 3-membered rings |
| hbd | H-bond donor atoms | r4 | 4-membered rings |
| neg | negative charges | r5 | 5-membered rings |
| pos | positive charges | r6 | 6-membered rings |
|  |  | r7 | 7-membered rings |
|  |  | r8 | 8-membered rings |
|  |  | r9 | 9-membered rings |
|  |  | rg10 | ≥ 10 membered rings |
|  |  | afr | atoms shared by fused rings |
|  |  | bfr | bonds shared by fused rings |

^a)^ Polarity counts consider the ionization state predicted for the physiological pH = 7.4. hbam counts lone pairs on H-bond acceptor atoms and hbdm counts H-atoms on H-bond donating atoms. ^b)^ All topology counts refer to the smallest set of smallest rings. afr and bfr count atoms respectively bonds shared by at least two rings.

**Table S2**. Composition of SMIfp

| no. | Symbol | Feature counted |
| --- | --- | --- |
| 1 | C | Non-aromatic carbon atoms |
| 2 | c | Aromatic carbon atoms |
| 3 | N | Non-aromatic nitrogen atoms |
| 4 | n | Aromatic nitrogen atoms |
| 5 | O | Non-aromatic oxygen atoms |
| 6 | o | Aromatic oxygen atoms |
| 7 | S | Non-aromatic sulphur atoms |
| 8 | s | Aromatic sulphur atoms |
| 9 | F | Fluorine atoms |
| 10 | Cl | Chlorine atoms |
| 11 | Br | Bromine atoms |
| 12 | I | Iodine atoms |
| 13 | P | Non-aromatic phosphorus atoms |
| 14 | p | Aromatic phosphorus atoms |
| 15 | B | Boron atoms |
| 16 | "X" | Any other element^a), b)^ |
| 17 | — | Explicit single bonds^c)^ |
| 18 | = | Double bonds |
| 19 | # | Triple bonds |
| 20 | [ | Special features^d)^ |
| 21 | - | Negative charges^b)^ |
| 22 | + | Positive charges^b)^ |
| 23 | H | Explicit hydrogen atoms^b), e)^ |
| 24 | ( | Acyclic branching points |
| 25 | 1 | Non-fused ring systems |
| 26 | 2 | Bicyclic systems |
| 27 | 3 | Tricyclic systems |
| 28 | 4 | Tetracyclic systems |
| 29 | 5 | Pentacyclic systems |
| 30 | 6 | Hexacyclic systems |
| 31 | 7 | Heptacyclic systems |
| 32 | 8 | Octacyclic systems |
| 33 | 9 | Nonacyclic systems |
| 34 | % | Higher order ring systems |

^a)^ includes 2-letter combinations. ^b)^ always within [ ]. ^c)^ always outside [ ]. ^d)^ non-organic elements, charges, isotopes, protonation states. ^e)^ on charged atoms. The following symbols are not considered: /, \, :, ., @.


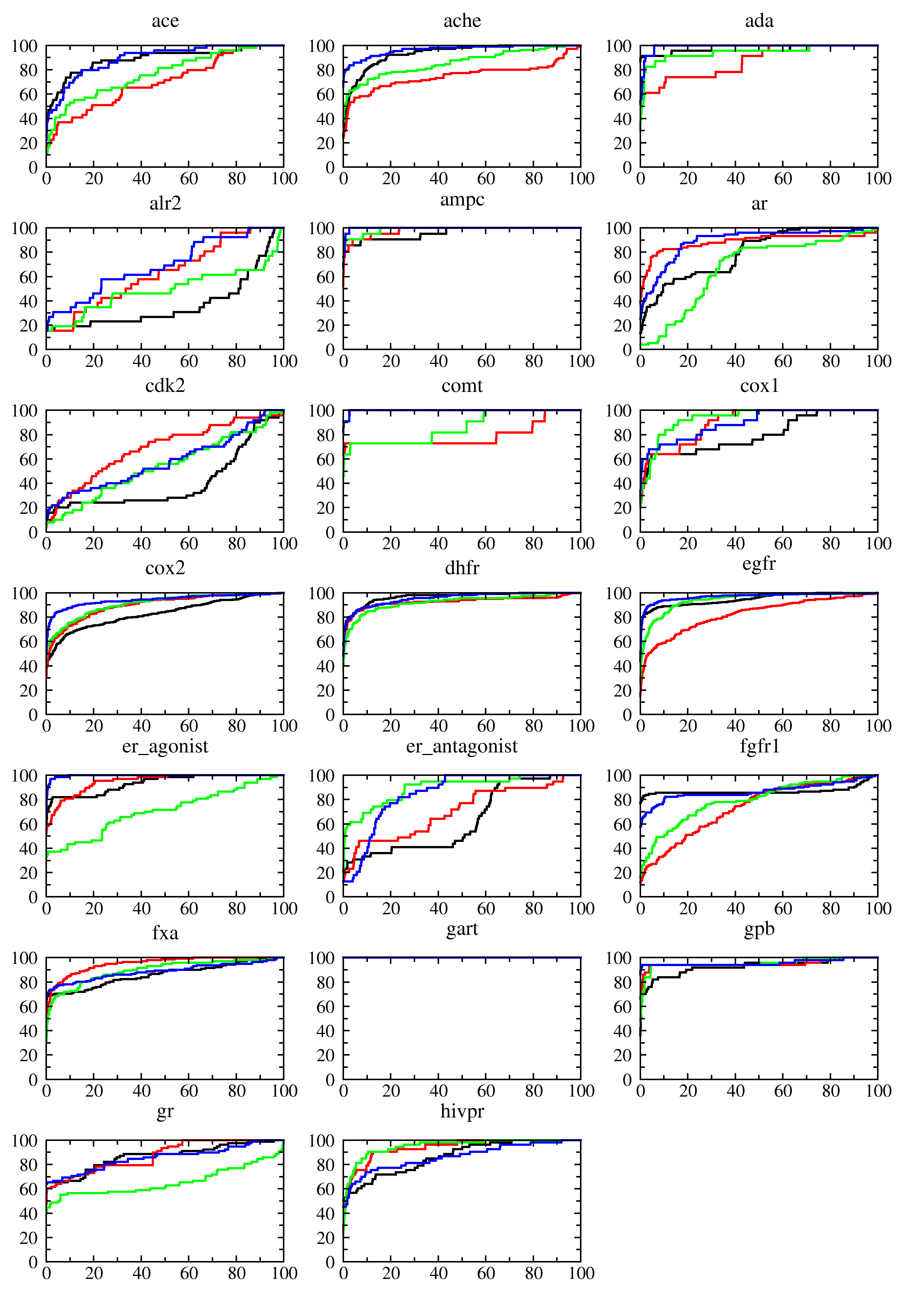


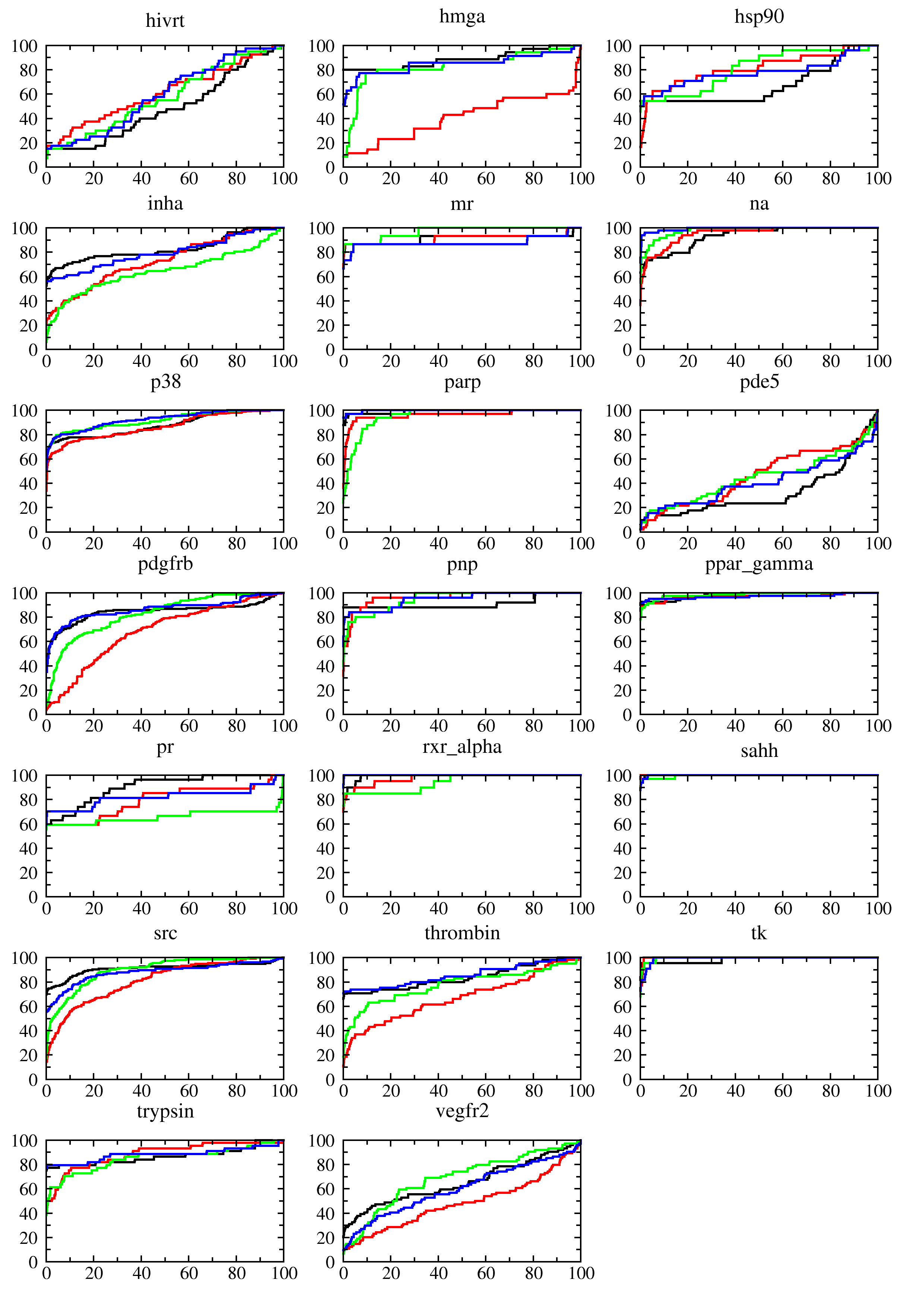


**Figure S1.** Receiver Operating Characteristic (ROC) curves for recovery of DUD actives from ZINC using sFP (black), ECFP4 (blue), MQN (red) and SMIfp (green). City block distance (CBD_fingerprint_) was used as scoring function. X-axis is % of sorted database and Y-axis=% of actives found.


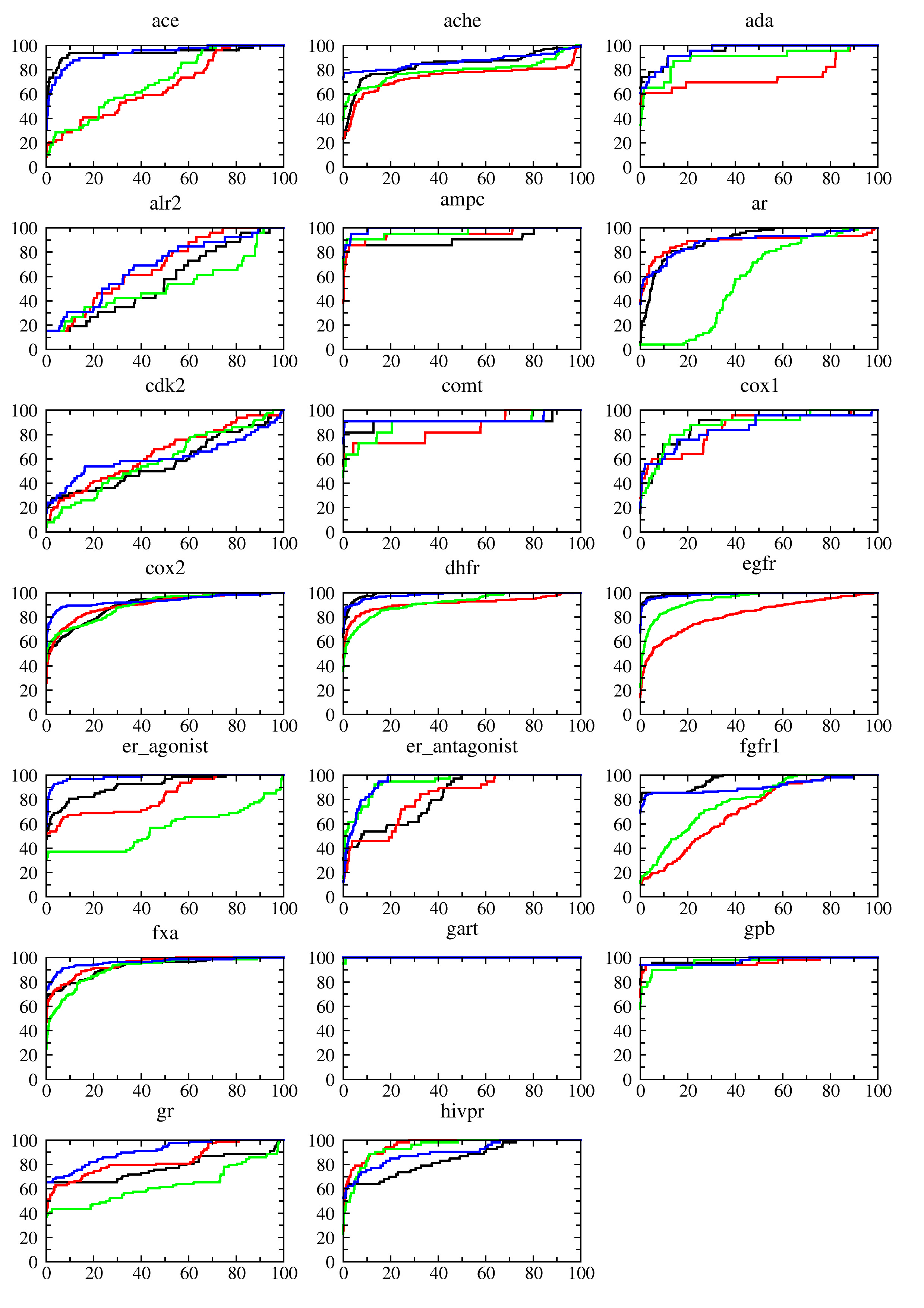


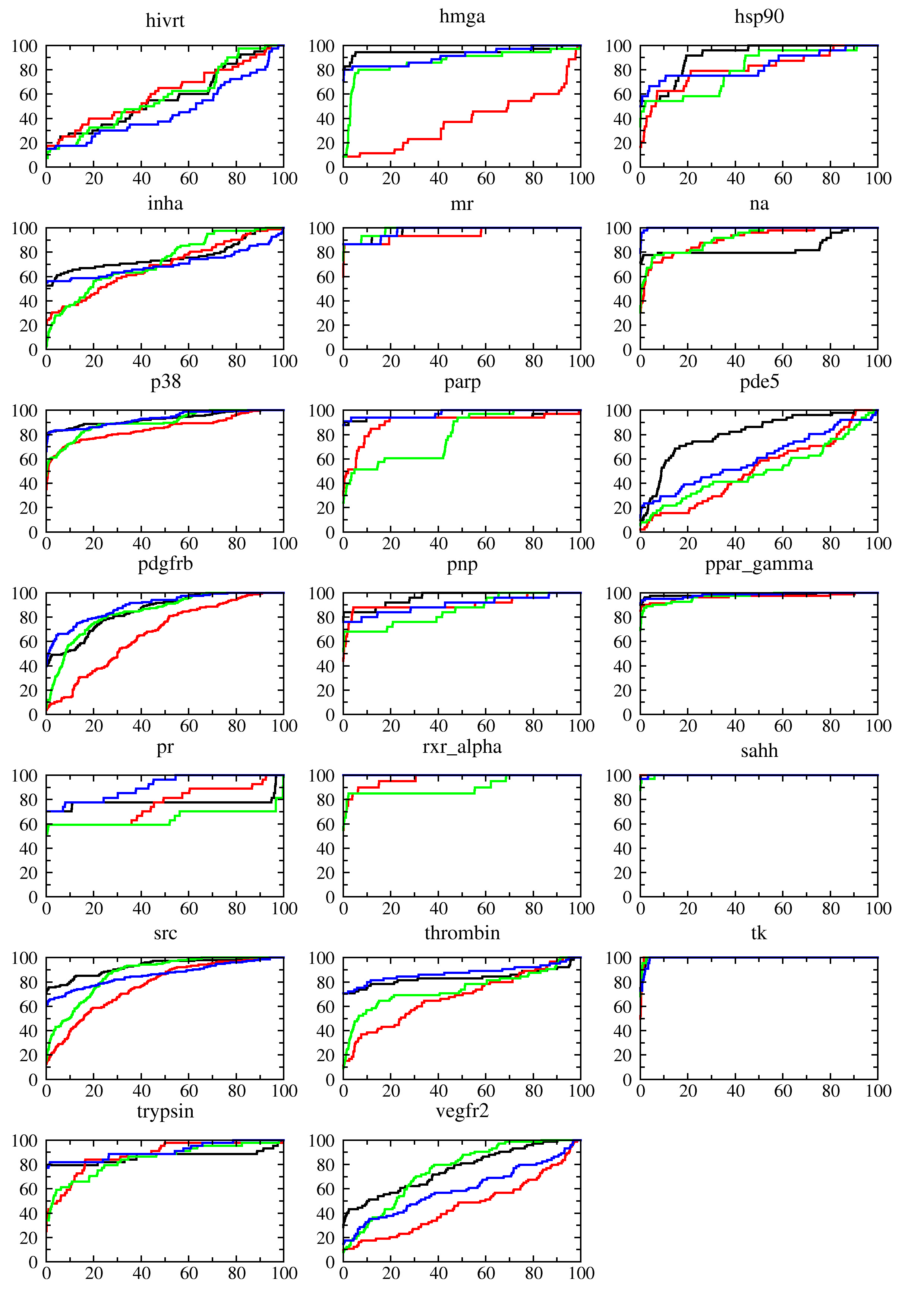
**Figure S2.** Receiver Operating Characteristic (ROC) curves for recovery of DUD actives from ZINC using sFP (black), ECFP4 (blue), MQN (red) and SMIfp (green). Tanimoto coefficient (*T*_fingerprint_) was used as scoring function. X-axis is % of sorted database and Y-axis=% of actives found.

**
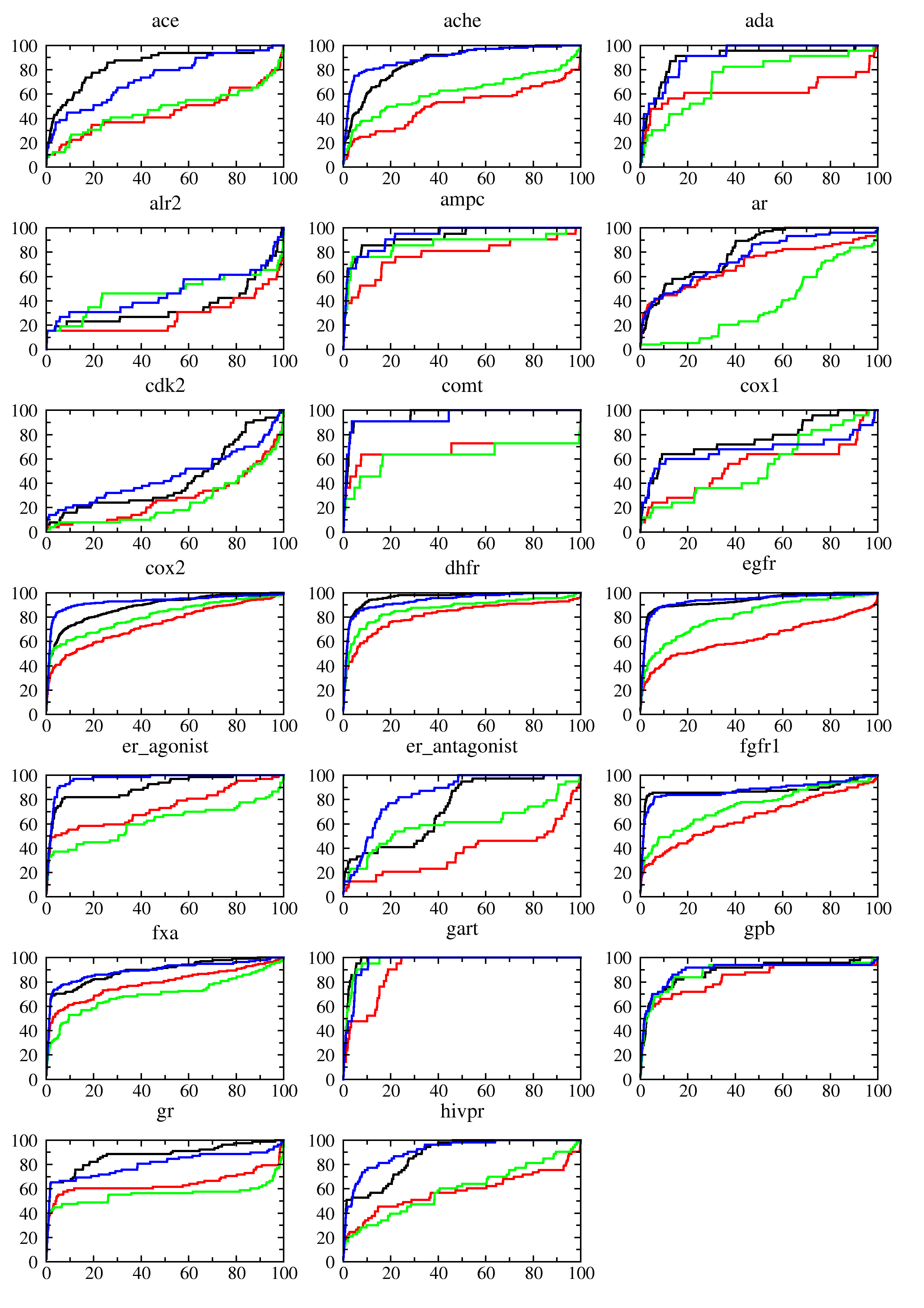
**


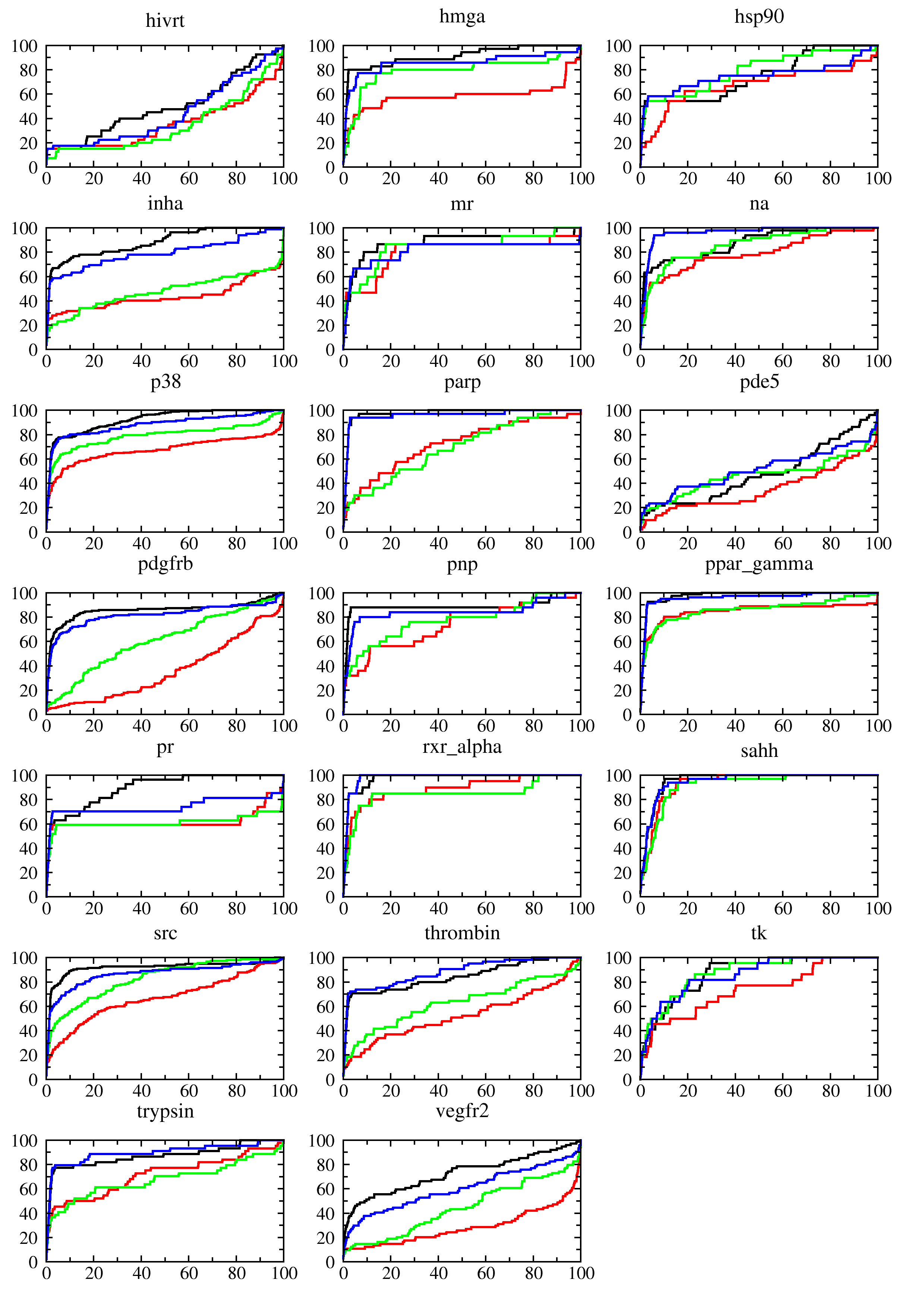
**Figure S3.** Receiver Operating Characteristic (ROC) curves for recovery of DUD actives from decoys using sFP (black), ECFP4 (blue), MQN (red) and SMIfp (green). City block distance (CBD_fingerprint_) was used as scoring function. X-axis is % of sorted database and Y-axis=% of actives found.

**
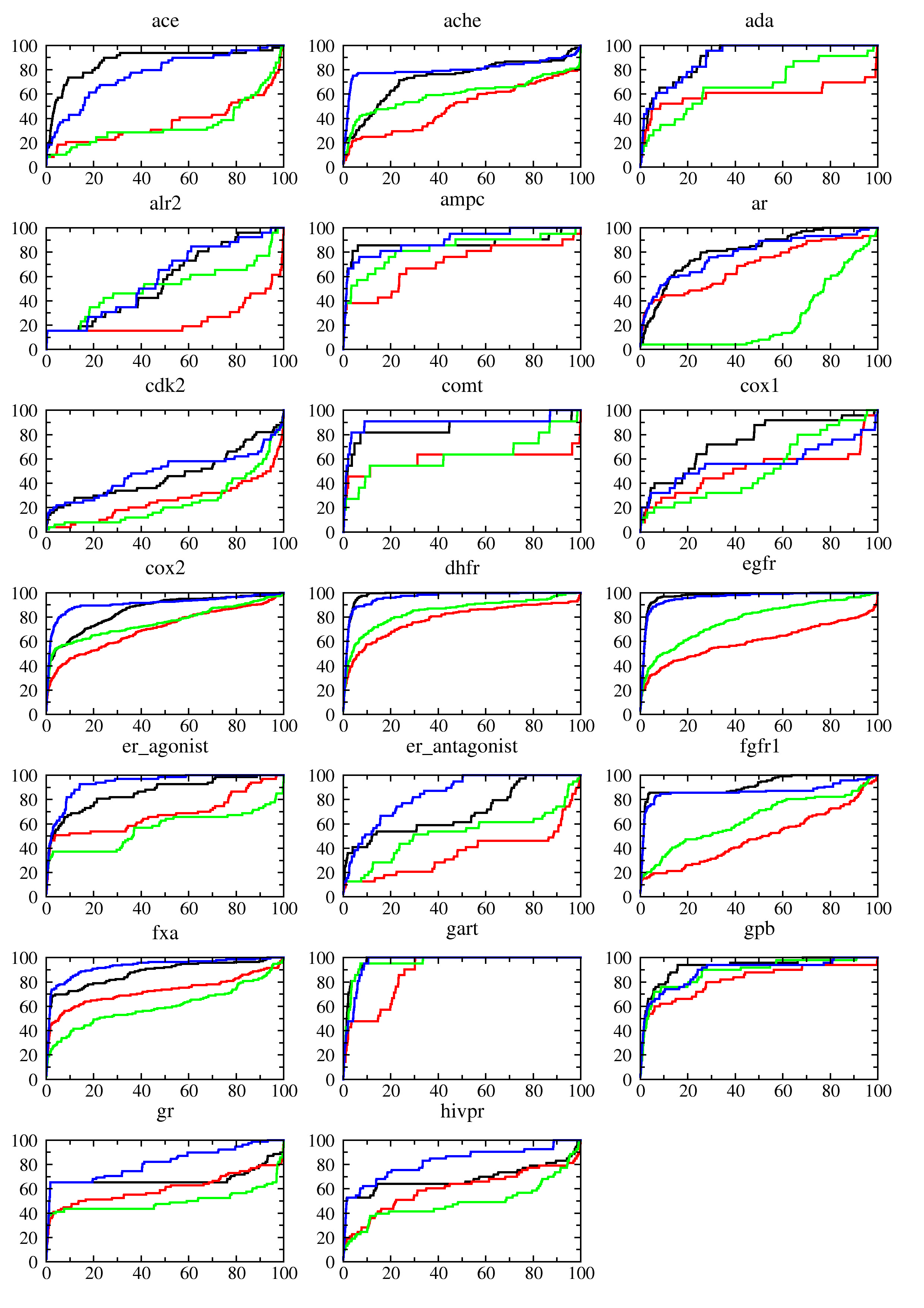
** **
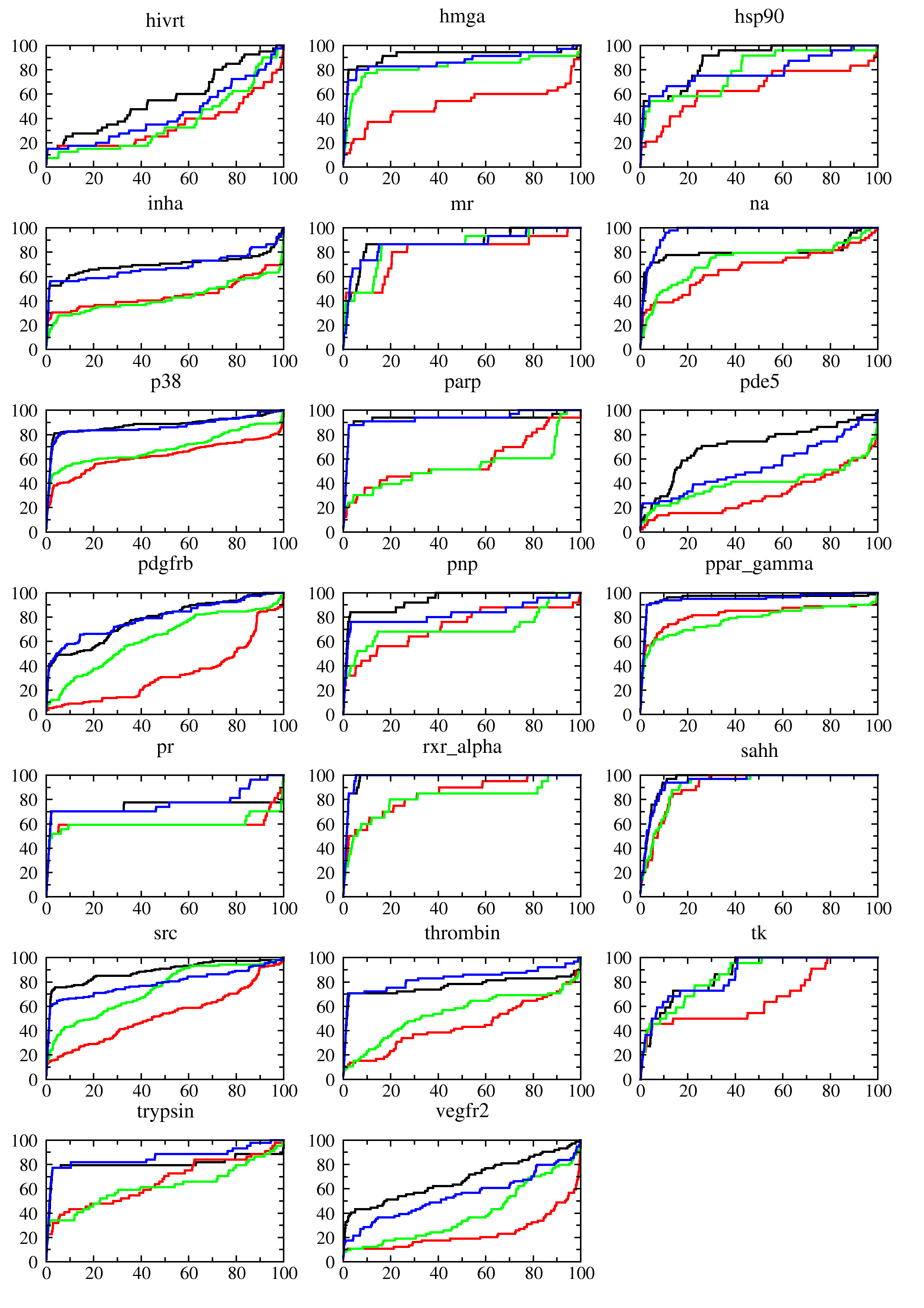
Figure S4.** Receiver Operating Characteristic (ROC) curves for recovery of DUD actives from decoys using sFP (black), ECFP4 (blue), MQN (red) and SMIfp (green). Tanimoto coefficient (*T*_fingerprint_) was used as scoring function. X-axis is % of sorted database and Y-axis=% of actives found.


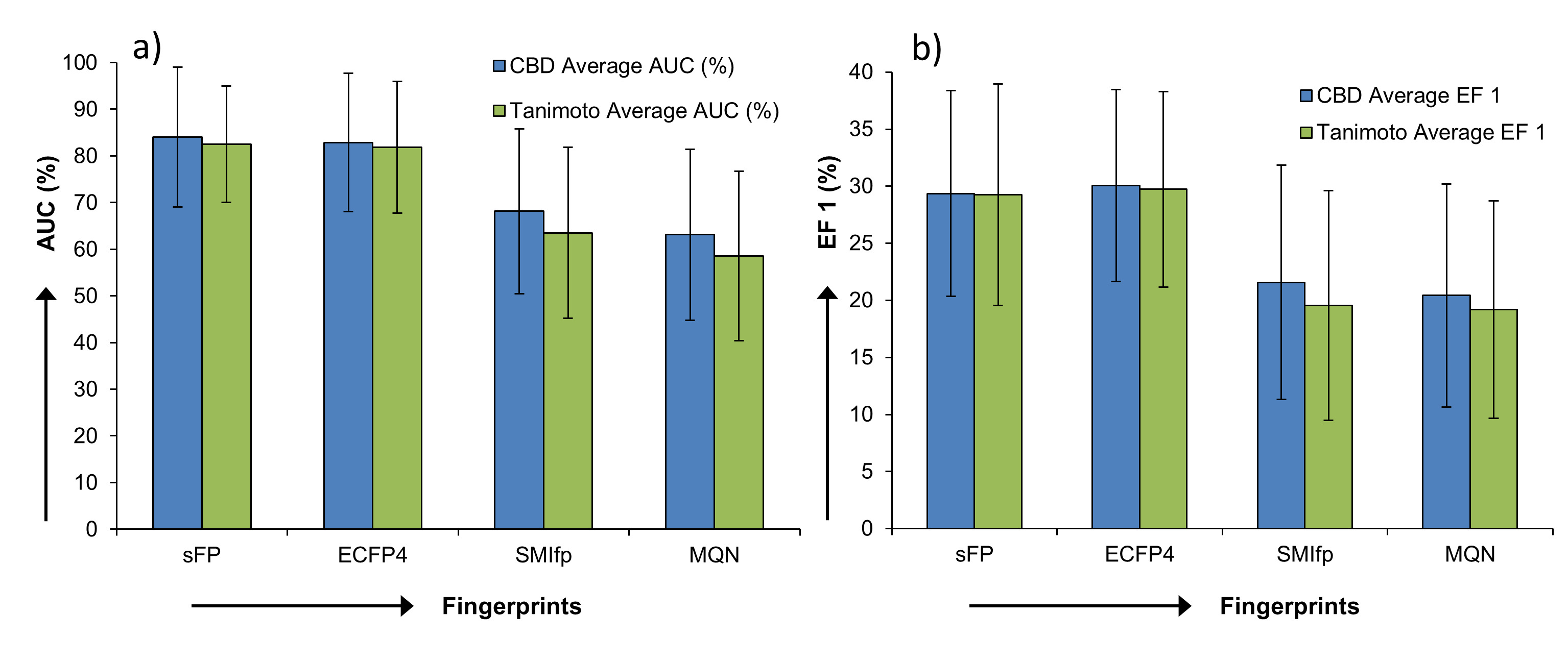


**Figure S5.** Average AUC values (a) and Enrichment factors at 1% of screened database (b), for recovery of 40 sets of actives in the directory useful decoys (DUD) from the corresponding decoys set by using CBD_fingerprint_ (blue bars) and *T*_fingerprint_ (green bars) as scoring functions. ROC curves are provided in Supplementary Figures S1-S2.
